# Determination of Femoral Neck Angle and Torsion Angle Utilizing a Novel Three-Dimensional Modeling and Analytical Technology Based on CT Datasets

**DOI:** 10.1371/journal.pone.0149480

**Published:** 2016-03-02

**Authors:** Maximilian J. Hartel, Andreas Petersik, Anne Schmidt, Daniel Kendoff, Jakob Nüchtern, Johannes M. Rueger, Wolfgang Lehmann, Lars G. Grossterlinden

**Affiliations:** 1 Department of Trauma, Hand and Reconstructive Surgery, University Medical Center Hamburg–Eppendorf, Germany; 2 Stryker Trauma GmbH, Kiel-Schönkirchen, Germany; 3 HELIOS ENDO-Klinik, Orthopedic Department, Holstenstr. 2, Hamburg, 20457, Germany; Le Fe Health Research Institute, SPAIN

## Abstract

**Introduction:**

Exact knowledge of femoral neck inclination and torsion angles is important in recognizing, understanding and treating pathologic conditions in the hip joint. However, published results vary widely between different studies, which indicates that there are persistent difficulties in carrying out exact measurements.

**Methods:**

A three dimensional modeling and analytical technology was used for the analysis of 1070 CT datasets of skeletally mature femurs. Individual femoral neck angles and torsion angles were precisely computed, in order to establish whether gender, age, body mass index and ethnicity influence femoral neck angles and torsion angles.

**Results:**

The median femoral neck angle was 122.2° (range 100.1–146.2°, IQR 117.9–125.6°). There are significant gender (female 123.0° vs. male 121.5°; p = 0.007) and ethnic (Asian 123.2° vs. Caucasian 121.9°; p = 0.0009) differences. The median femoral torsion angle was 14.2° (-23.6–48.7°, IQR 7.4–20.4°). There are significant gender differences (female 16.4° vs. male 12.1°; p = 0.0001). Femoral retroversion was found in 7.8% of the subjects.

**Conclusion:**

Precise femoral neck and torsion angles were obtained in over one thousand cases. Systematic deviations in measurement due to human error were eliminated by using automated high accuracy morphometric analysis. Small but significant gender and ethnic differences were found in femoral neck and torsion angles.

## Introduction

The angulation and torsion of the proximal femur in normal populations has been subject to research for decades. [1–12] Recognition, understanding and treatment of pathologic conditions in the hip joint must be supported by exact knowledge of the normal values of these parameters, especially in growing children, but also in skeletally mature patients. [2,13,14] For diagnosis and patient selection in derotational osteotomy, and for correction of rotation after femoral nailing, it is of the utmost importance to determine individual torsion angles precisely and to be aware of the distribution of angles in the normal population, as well as any ethnic differences. [9,15,16] This knowledge is also vital in the field of hip replacement surgery, as anatomic reconstruction of hip joint architecture plays an important role in attaining favorable functional outcomes. [17–19] Moreover, it may help to reduce the risk of bony impingement and impaired ROM due to the failure to retain femoral anterior offset in total hip arthroplasty. [20]

Researchers have described femoral anatomy in cadavers [5,7,10,12] and have used techniques including plain radiography [1,5,11], computed tomography [7,9], 3D-reconstructed computed tomography [14] and MRI [21]. Although extensive research has been performed, results vary over a comparatively large range, which indicates that there are persistent difficulties in carrying out exact measurements. The variance of results may partly be explained by scientific progress over time. However, Davids et al could show that measurement accuracy is compromised by femoral specimen malalignment even if three-dimensional volumetric CT images were used under laboratory conditions. [22] This finding indicates that widely established software solutions and calculation models might still lack the accuracy needed to precisely determine anatomic angles at the proximal femur. In the present study, a novel three-dimensional modeling and analytical technology (Stryker Orthopaedics Modeling and Analytics system, SOMA) was applied to a large CT database. With this technology, a highly refined measuring methodology could be used, in order to obtain true individual femoral neck angles and torsion angles in normal populations. The technology is capable of automatically determining femoral neck angles (inclination) and torsion angles. Poor inter-observer agreements in torsion angle analyses is then no longer a problem. [23,16] Updated reference values for femoral neck angles and torsion angles are presented. The influence of gender, age, body mass index and ethnicity on femoral neck angles and torsion angles was analyzed.

## Methods

**Fig 1:**
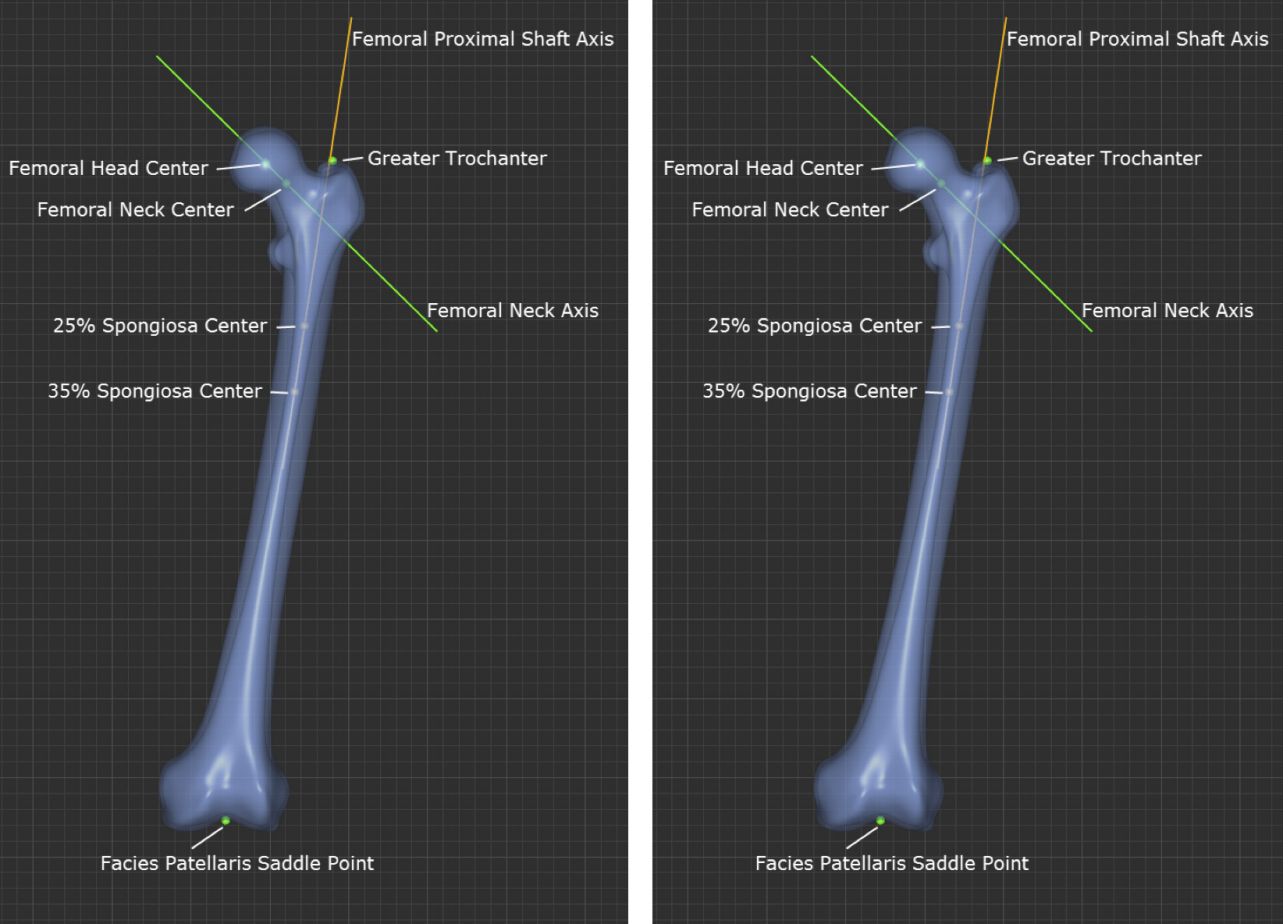
Fig 1 shows the definition of “femoral proximal shaft axis” and “femoral neck axis” with (a) AP View, (b) LM View.

SOMA was originally developed in collaboration between the Clinic of Orthopedics and Sports Orthopedics of the Technical University of Munich, Germany, and Stryker Trauma GmbH (Kiel, Germany). All CT scans were acquired exclusively for medical indications: Polytrauma (20%), CT angiography (70%), and others (10%). As previous work has shown that there is significant individual symmetry when paired femurs are compared, only left femurs were used for this analysis.[24] A total of 1070 thin-slice CT datasets of left femurs were analyzed in a cross-sectional study, using the SOMA custom software (Pixel spacing: Median: 0.78 mm, IQR: 0.14 mm, Slice Spacing: Median: 1.00 mm, Interquartile Range: 0.20 mm). See also S1 and S2 Files for further information). Femurs with fractures, deformities, and hardware in situ were excluded. To permit the use of datasets in SOMA, which relies on 3D-bone datasets, all CT-scans were segmented with standard software (MeVisLab and Materialise Mimics), according to a standardized protocol. SOMA automatically transferred measurements defined on an averaged 3D-bone template based on the available 1070 datasets to each dataset. (Fig 1) This ensures highly accurate and reproducible measurements across a large population. [23] It is possible to measure lengths, angles and diameters based on points, lines, planes and circles.

### Determination of the axes of the femoral shaft and neck

The definition of the axes of the femoral shaft and neck is critical for correct three-dimensional measurement of femoral neck angle.

Since the femur exhibits considerable curvature in three dimensional space, the axis of the femoral shaft cannot be defined along the whole length of the femur, as it would run outside the bone in the middle third of the diaphysis in many individuals. This study focuses on the anatomic characteristics of the proximal third of the femur. Therefore the proximal femoral shaft axis was defined by connecting the inner cortical midpoints at 25% and 35% of bone length (measured from the tip of the greater trochanter to the facies patellaris saddle point). In selecting 25% and 35% of bone length, care is taken that the axis definition starts well below the lesser trochanter, as previous work has shown that there is significant inter-individual variance in the inner cortical shape in this area and that the line definition ends at the transition between the proximal and middle thirds of the femur. [25]

To precisely determine the axis of the femoral neck, the femoral head center was automatically calculated. The location of the isthmus of the femoral neck and the centroid of the inner cross-sectional area were then computed. The resulting two points connect to give a true femoral neck axis. Fig 1

### Determination of femoral neck angles and torsion angles

To obtain the femoral neck angle, the angle between the femoral neck and the shaft axis was automatically calculated for each subject, using the technology described above.

**Fig 2:**
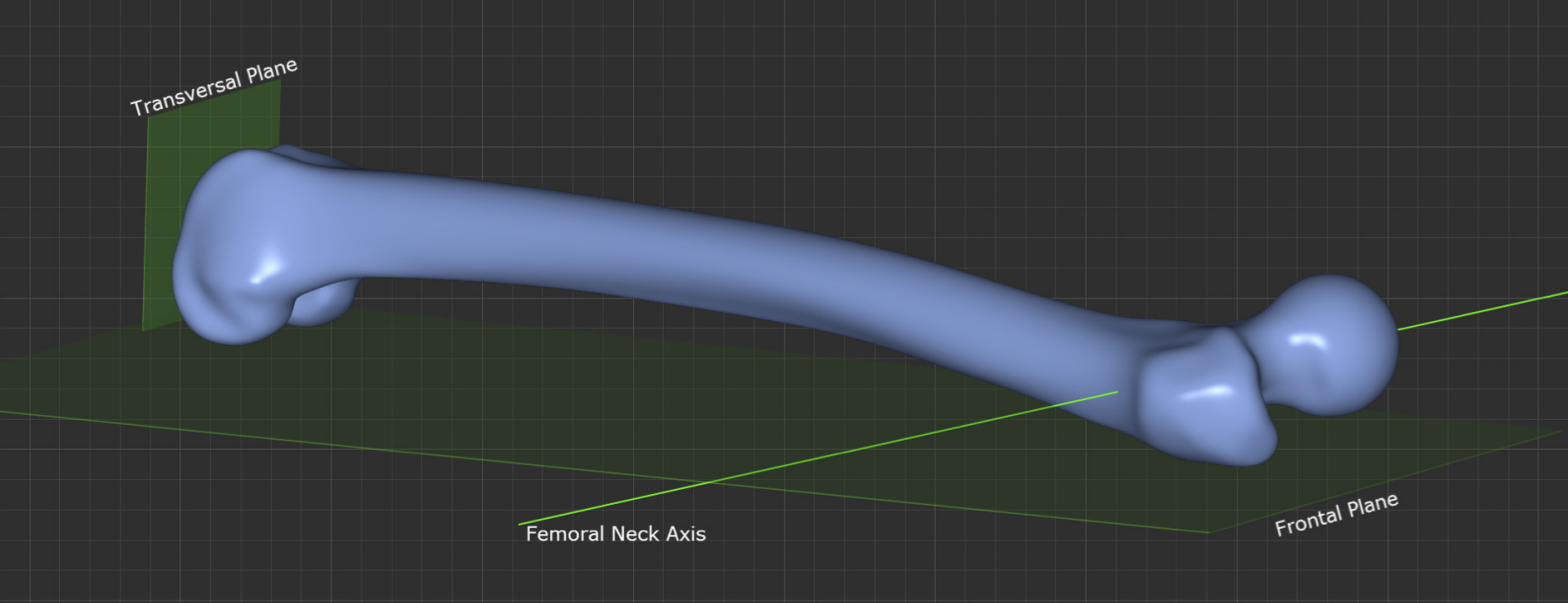
Fig 2 shows the definition of frontal and transversal plane.

**Fig 3:**
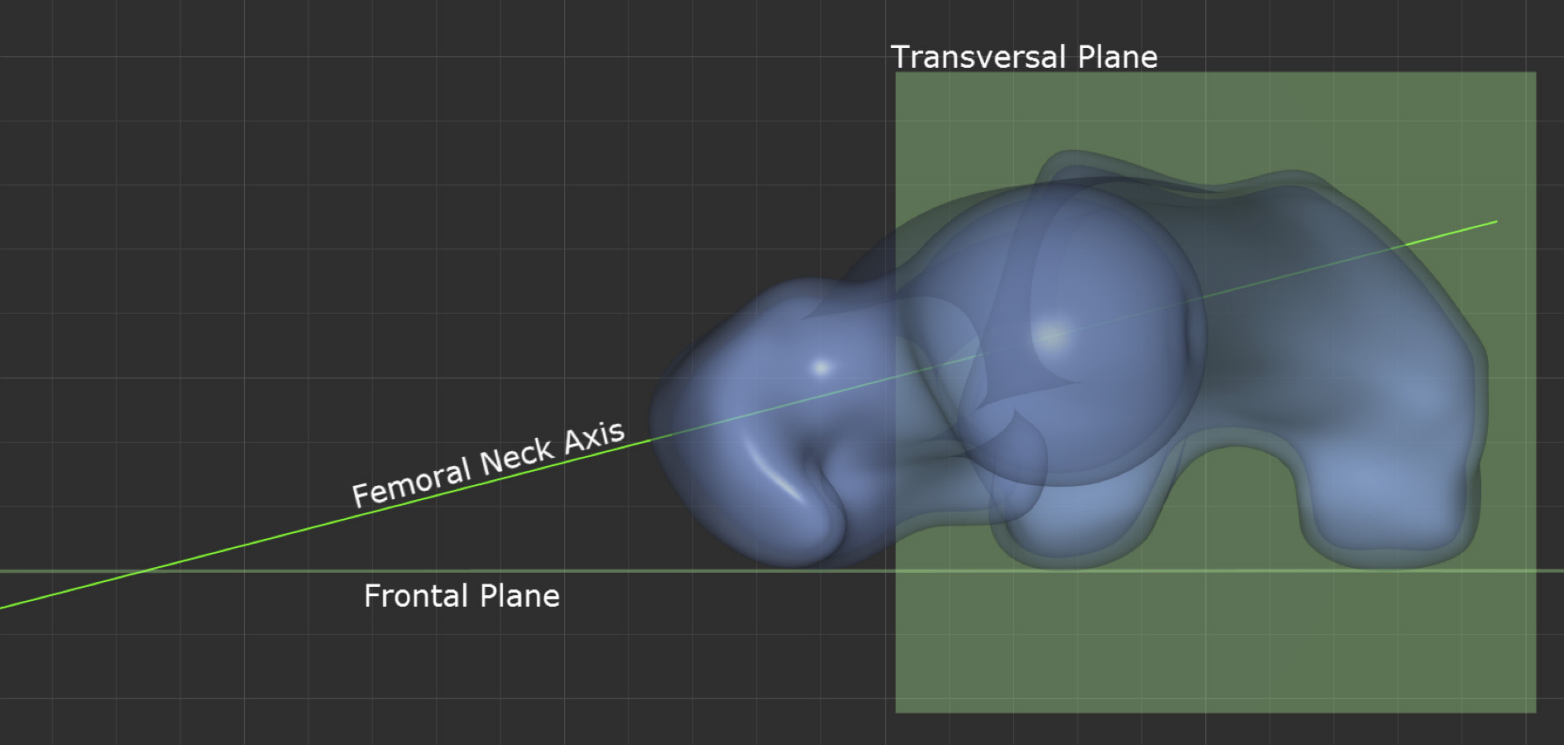
Fig 3 shows the measurement of femoral torsion: Angle between frontal plane and femoral neck axis projected onto transversal plane.

Several methods have been described for determining the condylar line at the distal femur. The most commonly used method is the “table top method”. A tangent line is drawn between the posterior peaks of the femoral condyles. [2,3,7,12] Previous research has shown that this method provides the best reproducibility.[9] This method was not modified, in order to ensure comparability with previously published data. Fig 2 shows the definition of the frontal and transversal plane. The femoral neck axis is projected onto the transversal plane and the acute angle between the projection and the table top posterior intercondylar tangent line corresponds to the femoral torsion as Fig 3 shows.

### Relevant Parameters

It was analyzed how gender, age, body mass index and ethnicity influence femoral neck angles and torsion angles.

### Statistics

Continuous parameters were reported as medians (interquartile range). Categorical variables were reported as absolute counts (percent). As some of the collected variables followed a non-normal distribution, statistical analyses always employed nonparametric approaches. Spearman correlations were therefore calculated for associations between continuous variables. The Wilcoxon test and the Mann–Whitney U test are used to compare continuous variables between paired or independent groups.

All statistical analyses were conducted using IBM SPSS V21 (IBM Corp., NY, USA).

## Results

In all, 1070 CT datasets of skeletally mature left femurs were examined. There were 810 femurs of Caucasian (76%), 27 of African (3%), 217 of Asian (20%) and 15 of Middle Eastern (1%) origin. Because of the lower numbers, the subjects originating from the Middle East and Africa were left out of the sub-analyses for ethnic differences, leaving a total of 1027 for this part of the investigation. Forty-five percent (45%; n = 485) of the subjects were female. The median age was 65 (n = 983, range 19–109, IQR 52–74). Data on height and weight for the calculation of the body mass index (BMI) were available in 799 (75%) of the subjects. The median BMI was 25.7 (n = 799; range 11.7–54.7, IQR 23.1–29.0). See also Table 1 for a summary of femoral neck and torsion angles found the cohort.

**Table 1:** Provides a summary of femoral neck and torsion angles found the cohort.

	All femurs	Female	Male	Asians	Caucasians
Femoral neck angle	122.2°	123.0°	121.5°	123.2°	121.9°
[median°]	(range 100.1–146.2°, IQR 117.9–125.6°)	(range 100.1–146.2°, IQR 118.9–127.0°)	(range 105.3–138.2°, IQR 118.1–125.6°)	(range 110.2–138.6°, IQR 120–126.6°)	(range 100.1–146.2°, IQR 118.1–126°)
Femoral torsion	14.2°	16.4°	12.1°	14.7°	14.2°
[median°]	(range -23.6–48.7°, IQR 7.4–20.4°)	(range -19.9–48.7°, IQR 10.5–22.7°)	(range -23.6–42°, IQR 5.7–18.4°)	(range -13.1–48.7°, IQR 5.5–21.4°)	(range -23.6–45.7°, IQR 8.1–20.3°)

### Femoral neck angles

**Fig 4:**
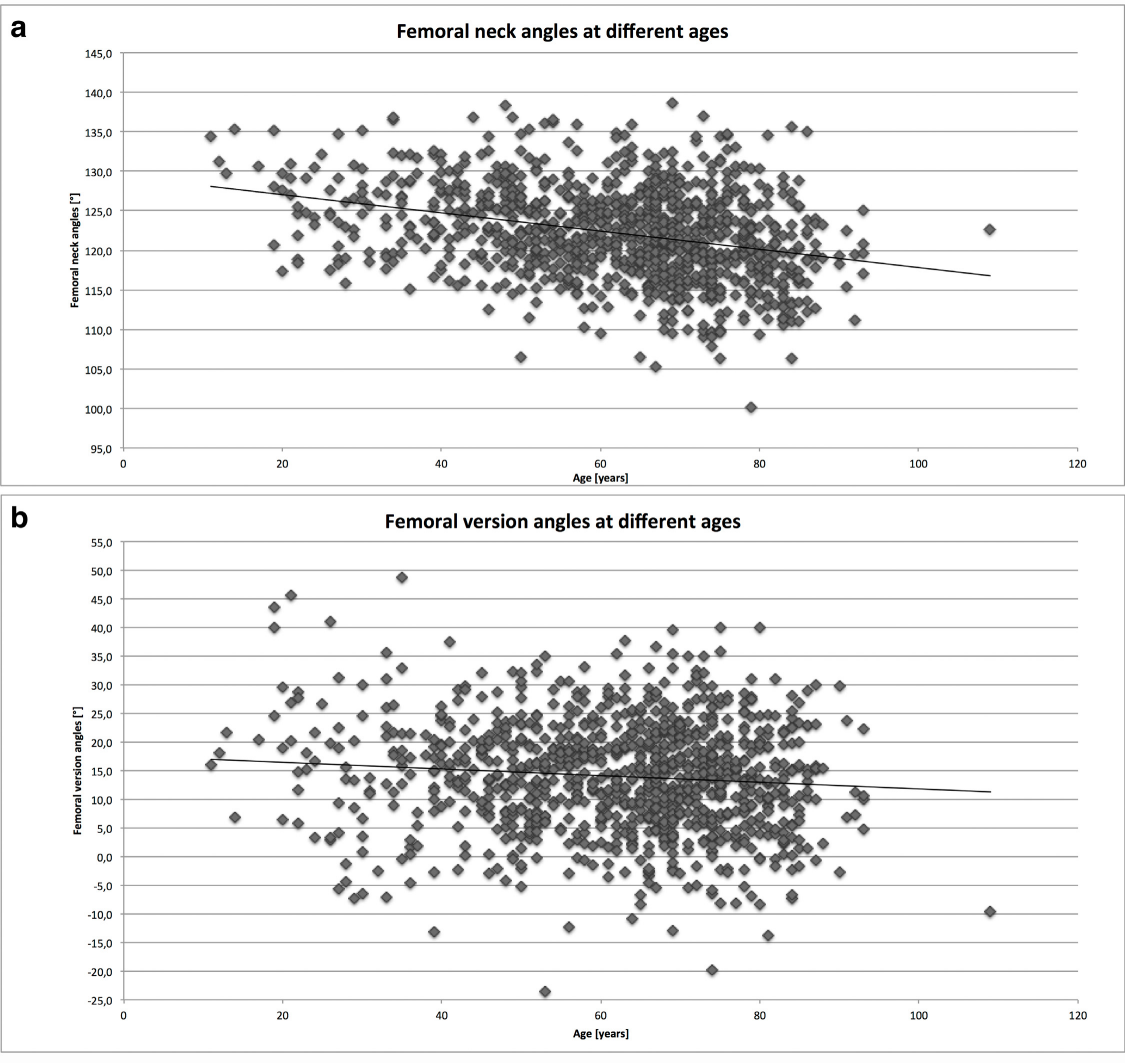
Fig 4a depicts the femoral neck angles at different ages and Fig 4b depicts the femoral torsion angles at different ages.

The calculated median femoral neck angle was 122.2° (range 100.1–146.2°, IQR 117.9–125.6°). There is a significant gender difference (p = 0.007, female 123.0° vs. male 121.5°). The median neck angle for Caucasians was 121.9° (100.1–146.2. IQR 118.1–126.0°); the median neck angle for Asians was 123.2° (110.2–138.6°, IQR 118.1–126.0°). The difference between femoral neck angles for Caucasians and Asians is significant (p = 0.0009). No correlation was found between BMI and the femoral neck angles (r = -0.066; p = 0.06). As shown in Fig 4A, femoral neck angles tend to decrease in older subjects. Correlation testing confirms this inverse correlation (Spearman’s rho -0.31; p<0.0001). See also Table 1 for a summary of femoral neck and torsion angles found the cohort.

### Torsion angles

The calculated median femoral torsion angle was 14.2° (range -23.6–48.7°, IQR 7.4–20.4°). The medians differed significantly between the genders (female 16.4° IQR 10.5–22.7° vs. male 12.1°, IQR 5.7–18.4°; p = 0.0001). The median torsion angle for Caucasians was 14.2° (IQR 8.1–20.3°); the median torsion angle for Asians was 14.7° (5.5–21.4°). The difference was not significant (p = 0.8488). Correlation between the torsion angle and BMI just achieved statistical significance (Spearman’s rho -0.07; p = 0.054). The torsion angle decreased significantly with age, although the effect was minor. This effect was more marked in a subgroup analysis of 19–50 (n = 224) versus 75–109 year old (n = 220) patients (young 15.3°, IQR 8.0–21.3° vs. old 13.0°, IQR 6.3–19.6; p = 0.0155). Fig 4B shows the data distribution with a trend line. A wide range of torsion angles can be observed. See also Table 1 for a summary of femoral neck and torsion angles found the cohort.

### Femoral retroversion

There were 77 subjects (7.8%) with retroverted hips (range -23.6–0.2°, IQR -6.6–2°). The frequency of retroversion was not clearly influenced by ethnic origin (Caucasian 73%, Asian 21%, African 5%, Middle Eastern 1%). Men were represented more predominantly, with 52 cases (68%). Interestingly, patients with retroverted hips had a significantly higher BMI (normal 25.6, IQR 23.0–28.7 vs. retroverted 27.1 IQR 24.5–29.4; p = 0.0023).

## Discussion

Precise femoral neck and torsion angles were obtained using a database with 1070 CT datasets of left femurs. A novel three-dimensional modeling and analytical technology was used for the analyses. The truly new element in this study is that the process of data procession was entirely automatic. It has been demonstrated that such automated morphometric analyses are highly accurate: In a validation study, this technology showed a deviation of generally less than 2 mm when compared with a mean of manually entered landmarks (twelve medical experts, measurements repeated three times). [23] It could be concluded, that the “algorithmically detected correspondences are closer to the “gold standard” position than those obtainable from an average human.”

In the large population investigated, small but significant gender differences were found in femoral neck and torsion angles. There was also a small but statistically significant difference in femoral neck angles between Caucasian and Asian femurs. In earlier series, these findings were not reported, probably due to smaller sample sizes. [12,26] Over all, body mass index did not have a relevant impact on the angles measured. This finding confirms previously published results. [27] However subjects with retroverted hips were significantly more obese than subjects with neutral or anterior torsion.

Femoral retroversion was found in 8% of the population investigated. Koerner et al. found markedly higher proportions of retroverted hips in their population, ranging from 23.5% in African American females to 7.2% in Hispanic males. [26] This difference is probably methodological: In Koerner’s study, a 2D-CT model was used.

In order to obtain a standard characterization of the English thigh bone, Professor F. G. Parsons investigated a “the bones of medieval English people”. [10] He reported markedly higher mean femoral neck angles of 126° in males and 125° in females. The torsion angles differ less pronouncedly, with mean values of 13° in males and 16–18° in females. It is evident that comparibility with the current study is limited, as there were significant methodological differences. However, to some extent these differences may also be explained by a steady change in anatomical characteristics over the centuries and by differences in the quality of nutrition between now and then. In their study, Toogood et al. investigated thigh bones in an osteologic collection from “approximately at the beginning of the 20^th^ century”. [12] In this study, markedly lower torsion angles—with a mean of 9.73 ±9.28°—were obtained than in the current investigation (14.0° ±9.7°). Likewise, the femoral neck angles differ significantly (Toogood: 129.23 ±6.24° vs. present study: 122.3 ±5.83°). The methods used in these studies differ, as Toogood et al. took standardized photographs of cadaveric specimens and made the measurements on the digital photographs. However, if the results are compared with recent work using 3-dimensional computed tomography, significant differences are also found: Buller et al. found mean torsion angles of 4.7° in left (4.5° right) hips compared to the values of 14.0° ±9.7° in the present study. Similarly, different neck angle values were reported. [14] As the reference points underlying the measurements are very similar, this discrepancy may be due to individual variations in measurement. In conventional CT imaging, the most precise way to define the axis of the femoral neck, used summated axial CT cuts or both multiplanar and three dimensional reconstructions. In every case however the necessary measuring landmarks needed visual identification, using best fit circles and lines drawn by hand on the computer screen.[2] Moreover, these measuring procedures need to be repeated in hundreds of cases. Therefore, inter-individual proneness to error may be a problem. Yoon and colleagues demonstrated that the inter-observer agreement in torsion angle measurements is at best moderate in well trained attending physicians, with a Pearson’s r of 0.661 (p < 0.01) and at its worst in first year orthopedic residents, where there is almost no correlation. [16] One advantage of this study is probably the significant reduction in measurement errors, as there was no necessity to manually draw axes and circles. Using the novel three dimensional analysis technology, it was possible to automatically calculate the precise femoral neck axis.

Plain radiographs, CT-slides and even multiplanar reconstructed CT-views give a simplified impression of complex three-dimensional anatomic structures, such as the human femur. A three-dimensional image is therefore probably the best approach to obtain exact measurements in such structures.

Another advantage of the present study is the comparably high number of cases. More than a thousand femurs were included in this investigation.

The decrease in the femoral neck angle during lifetime, especially during growth, is well known. Moreover, a significant reduction in femoral torsion angles during growth has been documented. [28] In this study, smaller individual torsion and femoral neck angles tended to be observed in older skeletally mature individuals. It may be assumed that both the femoral neck angle, and the femoral torsion angle change throughout life.

One limitation of the age-related analyses is certainly that only inter-individual age-related differences could be collected and analyzed. More femoral neck and torsion angles at several times in an individual lifetime would be needed to achieve greater precision in such an analysis. However, to our knowledge, such detailed data are currently not available. Another limitation is that the available database lacks a sufficient number of subjects of African and Middle Eastern ethnicity. Generalization is therefore limited to some extent. Finally information about the extent of degenerative hip diseases was not available. Although such femurs with fractures, severe deformities, and hardware in situ were excluded, minor osteoarthritic changes to the femoral head may have altered the original anatomy to some extent.

In conclusion, precise femoral neck and torsion angles could be obtained in over one thousand cases using an automated morphometric measuring process. Small but significant gender differences were found in femoral neck and torsion angles.

## Supporting Information

S1 File S1 File contains additional information on the data used in this paper. (DOC)

S2 File S2 File contains the underlying raw data used in this study. The data file contains a legend explaining the meaning of each data column.
(XLSX)
