# Supplementary material for: Determination of Femoral Neck Angle and Torsion Angle Utilizing a Novel Three-Dimensional Modeling and Analytical Technology Based on CT Datasets
Source: PLoS One. 2016 Mar 2;11(3):e0149480. doi: 10.1371/journal.pone.0149480 (PMC4775021; doi:10.1371/journal.pone.0149480)

Additional information on the data analyzed for “**Determination of femoral neck angle and torsion angle utilizing a novel three-dimensional modeling and analytical technology based on CT datasets**“

Dear Editors,

Dear Reviewers,

Please find in the following some additional information concerning the data used in this paper.

Yours sincerely

The Corresponding Author

The following two diagrams show the indication spectrum and origin of the CT datasets.


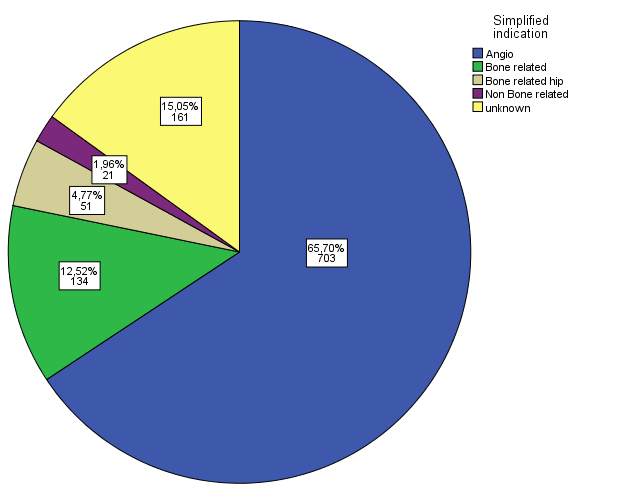


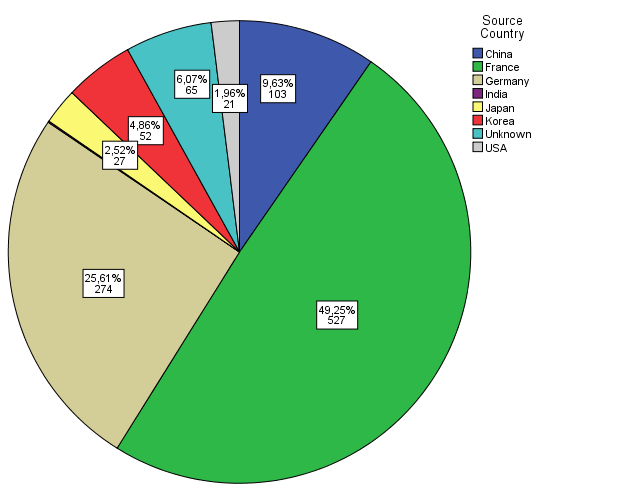


Technical details on the CT scans

Scanning resolution: Pixel Spacing: Median: 0.78 mm, Interquartile Range: 0.14 mm

Slice Spacing: Median: 1.00 mm, Interquartile Range: 0.20 mm


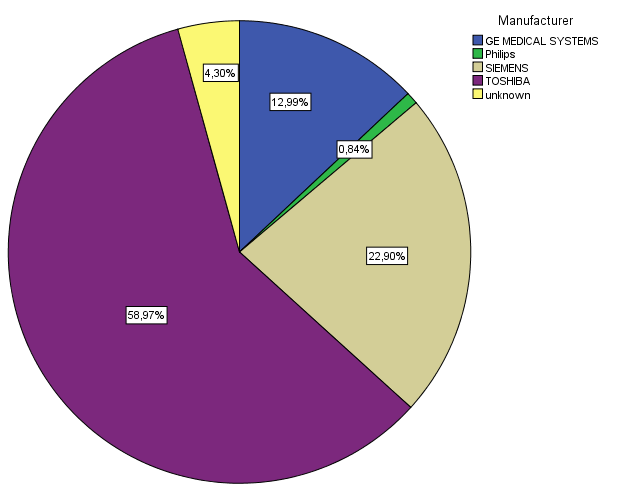

Supplement: S1 File — (DOC) [file pone.0149480.s001.doc]
